# Flow Process for Ketone Reduction Using a Superabsorber-Immobilized Alcohol Dehydrogenase from *Lactobacillus brevis* in a Packed-Bed Reactor

**DOI:** 10.3390/bioengineering6040099

**Published:** 2019-10-24

**Authors:** Niklas Adebar, Harald Gröger

**Affiliations:** Chair of Industrial Organic Chemistry and Biotechnology, Faculty of Chemistry, Bielefeld University, Universitätsstr. 25, 33615 Bielefeld, Germany; niklas.adebar@uni-bielefeld.de

**Keywords:** alcohol dehydrogenase, enzyme immobilization, flow chemistry, process design

## Abstract

Flow processes and enzyme immobilization have gained much attention over the past few years in the field of biocatalytic process design. Downstream processes and enzyme stability can be immensely simplified and improved. In this work, we report the utilization of polymer network-entrapped enzymes and their applicability in flow processes. We focused on the superabsorber-based immobilization of an alcohol dehydrogenase (ADH) from *Lactobacillus brevis* and its application for a reduction of acetophenone. The applicability of this immobilization technique for a biotransformation running in a packed bed reactor was then demonstrated. Towards this end, the immobilized system was intensively studied, first in a batch mode, leading to >90% conversion within 24 h under optimized conditions. A subsequent transfer of this method into a flow process was conducted, resulting in very high initial conversions of up to 67% in such a continuously running process.

## 1. Introduction

The development of flow processes has gained tremendous interest in recent years in organic chemistry due to numerous advantages of such types of processes over classic batch approaches [1]. In addition to higher efficiency and safety advantages, the ability to ensure constant product quality has turned out to be a major driving force for the development of flow processes. In particular, for active pharmaceutical ingredients (APIs), this aspect is important, as the FDA along with the EPA, as the respective institutions in United States and Europe for approval of medicines, added aspects of continuous manufacturing to their guidelines [2,3], whereas for a long time the focus of flow processes was on “classic” chemical processes with, for example, hazardous and thermally labile substances and chemocatalytic reactions [4,5,6]. Recently, biocatalysis in flow processes has become an emerging field with an increasing number of examples [7,8,9]. An enzymatic reaction of particular interest for an application in flow mode due to its matured technology level is asymmetric ketone reduction, having a proven track record underlined by many successful examples demonstrated in the past for the batch mode [10,11,12,13]. Recently, significant process in the biocatalytic [14,15,16,17] reductive synthesis of alcohols starting from ketones, as well as syntheses by non-enzymatic means, have been reported [18,19,20,21]. In recent years, an increasing number of examples of biocatalysis in flow processes have been reported [22,23,24,25,26,27]. Although only being studied to a minor extent, some examples of ketone reductions with an alcohol dehydrogenase (ADH) in flow processes have been described [28,29,30,31,32,33,34]. For example, Wandrey’s and Liese’s groups studied in detail the application of enzyme membrane reactors for enzymatic ketone reduction in a continuously running mode [28,29], and packed bed reactor setups were described by further groups using carrier-bound ADH from *Lactobacillus brevis* (LB-ADH) in combination with a mobile aqueous phase [30], or magnetic nanoparticle-bound ADH [31]. In addition, Buehler et al. investigated intensively the use of ADH in aqueous/organic segmented flow systems. It was shown that emulsion formation could be eliminated, and enzymatic mass transfer-limited reactions can be enhanced [32]. Furthermore, different means of stabilization of this reaction system were investigated [33]. Very recently, our group published an improved downstream process by means of such a segmented flow process, illustrated using two different ADHs and substrates [34].

In continuous processes using packed bed reactors (PBR), catalyst immobilization is a prerequisite. Also, downstream processes benefit from immobilization, as separation of the reaction mixture from the catalyst is easier, and the catalyst reusability is improved [35]. Moreover, both, operational, as well as storage stability of the catalyst can potentially be significantly increased. However, often a significant loss of enzymatic activity during the immobilization process represents a drawback, and the reproducible production of immobilized catalyst may be a further challenge [36]. Numerous techniques have been developed over the past years, which can be classified in the following three major methods: (a) binding to a solid support (carrier), (b) entrapment (encapsulation) in polymer networks, or (c) cross-linking in the form of cross-linked enzyme aggregates (CLEAs) or cross-linked enzyme crystals (CLECs) [35]. Recently, much progress in the field of enzyme immobilization has been made [37,38,39,40,41]. 

In this work, the option of entrapment of enzymes in polymer networks has been chosen. Often for this purpose, organic polymers containing cavities, sol-gel-processed particles, or membranes are used. A special case is the use of superabsorbent polymers to immobilize the entire aqueous phase. [42,43]. In this case, the enzyme is immobilized in its native aqueous environment, whereby undesired interactions of protein moieties with the support can be prevented. Recently, Gröger et al. applied a method of immobilizing ADHs with superabsorber efficiently for the synthesis of diols in enantiomerically pure form [44]. In this example, the immobilized ADH from *Rhodococcus* sp. was used in organic solvents without a major loss in activity. Advantages of this method is the low cost, the very fast immobilization procedure (<30 min), and the excellent catalyst reusability. Challenging, in particular with respect to an application in a fixed bed reactor, might be the gel-like consistency of the hydrogel and mass transfer limitations through phase interfaces in general. 

**Figure 1:**
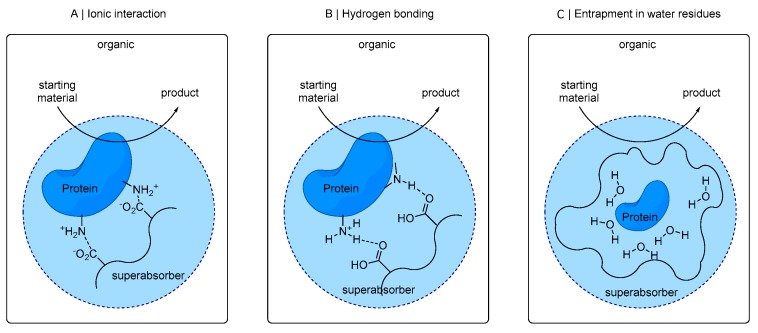
Scheme of superabsorber-based enzyme immobilization based on ionic interactions of acetate moieties of the superabsorber polymer particles and amino acid residues on the protein surface (**A**), hydrogen bonds of, for example, amino acids with carboxylic polymer residues (**B**), or entrapment of protein in water residues bound in the polymer matrix (**C**). Immobilized enzyme converts starting material dissolved in surrounding organic solvent to product, diffusing back into the organic solvent. The light blue circle with dashed line illustrates superabsorber particles with polymer network indicated as wavy lines in the surrounding organic solvent.

The absorbent polymer consists of cross-linked polyacrylic acid, which was partially neutralized [45]. Proteins can be embedded in this polymer matrix and can be bound, for example, by ionic interactions of its acid moieties to ionic amino acid residues of the protein (see Figure 1A). Also, hydrogen bridges from non-ionic protein residues with superabsorber functionalities (Figure 1B) or entrapment of enzyme in superabsorber-bound water residues (Figure 1C) can be a possible mode of interaction.

An (*R*)-enantioselective ADH from *Lactobacillus brevis* (LB-ADH) has been shown to be a versatile catalyst for the reduction of a variety of ketones, ranging from aliphatic ketones to benzylic or propargylic ketones [46,47]. It can be used as an isolated enzyme or as a whole-cell catalyst. In particular, its tolerance towards organic solvents is outstanding. The stability depends on several factors, such as the nature of the buffer or solvent additives [46,47].

In this contribution, the application of a superabsorber-immobilized ADH from *L. brevis* for an enzymatic reduction of acetophenone (**1**) in a flow process is described. 

**Figure 2:**
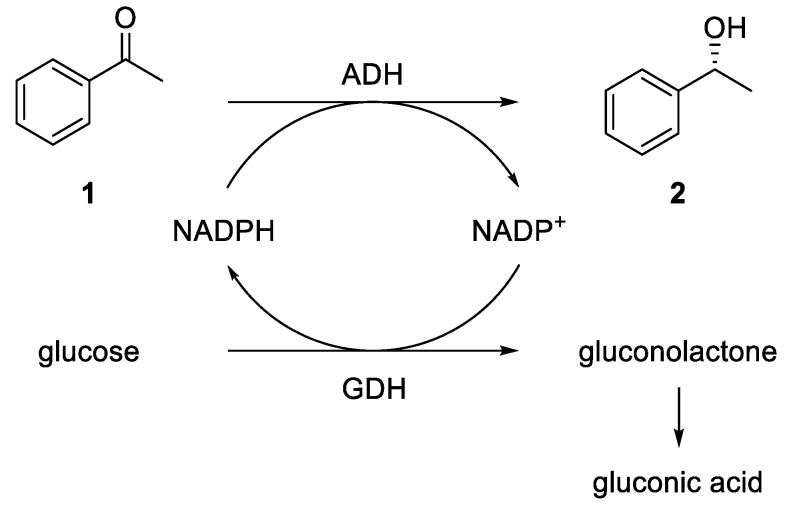
Alcohol dehydrogenase (ADH)-catalyzed reduction of acetophenone (**1**) to (*R*)-1-phenylethanol ((*R*)-**2**) using a glucose dehydrogenase (GDH)-based cofactor regeneration system.

Because the ADHs are cofactor-dependent enzymes and the cofactor NADPH is very expensive, an efficient cofactor regeneration system is required. In the literature, different options are described [48]. An elegant method is the use of isopropanol for an oxidation of the cofactor using the ADH itself. A further commonly used method is an enzyme cascade using an ADH and a glucose dehydrogenase (GDH). Here, the GDH reduces NADP^+^ back to NADPH, consuming one equivalent of glucose, which is oxidized to gluconolactone and irreversibly opens to gluconic acid in an aqueous environment (see Figure 2).

## 2. Materials and Methods

### 2.1. Preparation of the Recombinant Alcohol Dehydrogenase from Lactobacillus brevis

The preparation of recombinant LB-ADH overexpressed in *Escherichia. coli* has been previously described elsewhere, for example, in [46,47]. The recombinant LB-ADH for our study was kindly provided by Prof. Dr. Werner Hummel as whole cells. In order to obtain a crude extract of LB-ADH, the whole cells (4.5 g) were suspended in potassium phosphate buffer (6.8 mL, 0.1 M, pH 7, 1:3 (w/v)) and water (6.8 mL), then the resulting suspension was sonicated (5 × 2 min at 20%). After centrifugation (20,000 rpm, 4 °C, 30 min), the supernatant crude extract was decanted. The obtained crude extract was analyzed via photometer and Bradford assay, and aliquots were stored in the freezer at −20 °C for further use.

### 2.2. Characterization of the Recombinant Alcohol Dehydrogenase from Lactobacillus brevis

To determine the determination of biomass concentration, a standard Bradford assay was carried out as follows. Different dilutions of the samples were prepared (crude extract/buffer; 1:20, 1:30, 1:40). Reference (BSA (protein from *Bovines Serumalbumin* as standard) 1.4, 0.7, 0.35, 0.18, and 0.09 mg mL^−1^) and sample (5 µL) were pipetted in a microtiter plate, and Bradford reagent (250 µL) (see Table 1) was added. After incubation for 15 min at 25 °C while shaking, the absorption was measured at 550 nm. Three-fold determination was made for all measurements.

For the specific activity of the LB-ADH, a photometer assay was carried out. All photometer assays were carried out using a V-30 UV/VIS spectrophotometer from Jasco (Hachioji, Tokyo). According to Equation (1), the activity (in enzymeunits U mL^−1^) was calculated using the measured change in absorption *δA*, the total volume *V_tot_* (1 mL), the volume of crude extract *V_crude_* (10 µL), the molar attenuation coefficient of NADP^+^ at 340 nm *ε*_340_ (6200 L mol^−1^ cm^−1^), the pathlength *d* (1 cm), and the dilution *f*.

activity = *δA* × *V_tot_* × *f*/*ε*_340_ × *V_crude_* × *d*. (1)

In a photometer cuvette, buffer (446 µL), acetophenone solution (500 µL), and NADPH solution (24 µL) were mixed and blank measured. Then, ADH solution (30 µL) was added and the mixture was immediately measured at 20 °C and 340 nm after mixing (see Table 2). As described above, the enzyme activities (in U mL^−1^) were calculated from the cofactor absorption in the first 30–90 s.

### 2.3. Preparation of the Recombinant Glucose Dehydrogenase from Bacillus subtilis

Glucose dehydrogenase (GDH) from *Bacillus subtilis* was prepared according to the literature [49,50]. Enzymatic activity has shown similar activity, as reported in the literature. Aliquots of the obtained crude extract were stored at −20 °C until usage.

Further experimental data and procedures can be found in Appendix A.

## 3. Results and Discussion

### 3.1. Studies on the Comparison of Enzymatic Acetophenone Reduction in Biphasic Buffer/Methyl *tert*-Butyl Ether (MTBE) System versus Superabsorber/MTBE System in Batch Mode

At first, the LB-ADH-catalyzed reduction of acetophenone (**1**) to (*R*)-1-phenylethanol (**2**) was investigated in a biphasic system of methyl *tert*-butyl ether (MTBE) and buffer to set a benchmark for the immobilized enzymes. Initial investigations on influences of different parameters and optimization thereof were carried out in batch mode.

**Figure 3:**
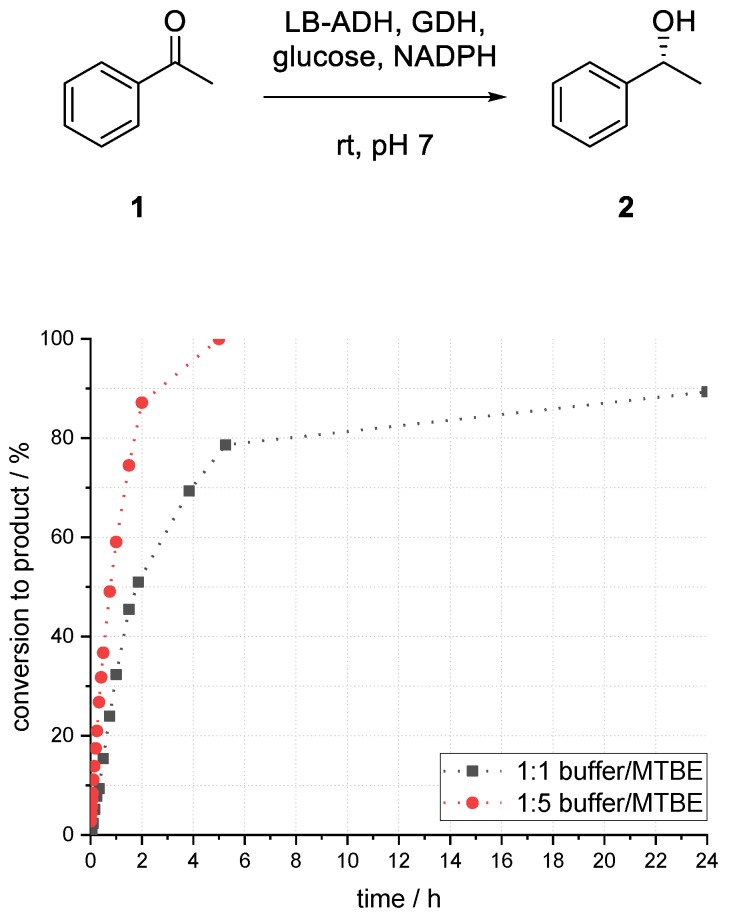
Biphasic ADH-catalyzed reduction of acetophenone (**1**) to 1-phenylethanol (*R*)-**2** in a buffer/ methyl *tert*-butyl ether (MTBE) system. c_substr_(overall): 50 mM, temperature: room temperature, catalyst loading: 2 U mL^−1^. LB: *Lactobacillus brevis*.

Compared to the reaction in buffer, which provided 86% conversion to alcohol (*R*)-**2** after 1.5 h, the biphasic reaction with a ratio of 1:1 (buffer/organic) provided only 45% (see Figure 3). After 24 h, the conversion to product (*R*)-**2** reached its maximum of 89%. In the case of a higher ratio of 1:5 (buffer/organic), a much faster conversion could be observed. After 1.5 h, 75% conversion to alcohol (*R*)-**2** and after 5 h full conversion could be obtained. Emulsion formation and formation of protein aggregates, sticking on the glass surface, could be observed within a short reaction time in all experiments by utilizing a biphasic reaction system.

**Figure 4:**
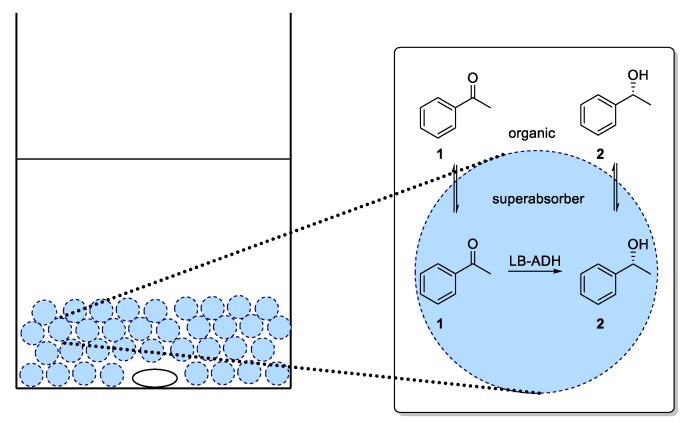
Schematic batch reaction of acetophenone (**1**) to alcohol (*R*)-**2** in a superabsorber system.

For an application of the ADH in a continuous process, immobilization of ADH utilizing a superabsorber was carried out. In this system, the enzymes are entrapped in an immobilized aqueous phase (as illustrated in Figure 4) and therein also cofactor and cosubstrate are present. In our studies, we exclusively focused on co-immobilization of both GDH and ADH. As diffusion of cofactor is crucial for an efficient process, immobilization of enzymes in different compartments being separated from each other was expected to be difficult in terms of cofactor availability. By means of a co-immobilization, such an effect could be minimized and the influence of process parameters on the ADH-catalyzed reduction could be studied. In the mobile organic phase, the substrate was dissolved. Thus, this type of immobilization enabled the enzymes to operate in their native aqueous environment, which also might lead to a sufficient stability when being used in such a superabsorber immobilizate.

**Figure 5:**
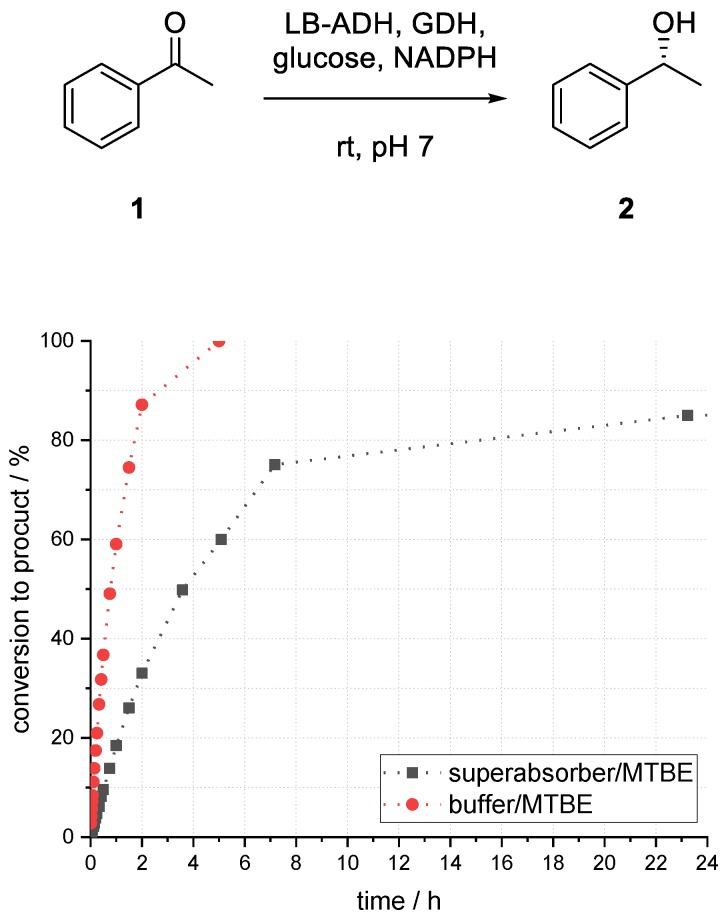
Comparison of enzymatic reduction of acetophenone (**1**) to phenylethanol (*R*)-**2** using ADH in biphasic buffer/MTBE system versus superabsorber/MTBE system, both 1:5 (v/v).

To gain a better understanding of this system, batch experiments screening different parameters were carried out. The conversion was monitored by gas chromatography (GC) analysis and the results after a reaction time of 25 h were compared. Different organic solvents, amounts of superabsorber, enzyme loadings, and buffer-to-organic solvent ratios were investigated. All aqueous solutions (ADH, GDH, NADPH, and glucose each in buffer) were mixed and immobilized by addition of superabsorber. After a short incubation of 5 min, a 100 mM acetophenone (**1**) solution in MTBE was added. All reactions were carried out at 25 °C. The results were compared to a buffer/MTBE system without immobilization (Figure 5).

Compared to a reduction using a biphasic buffer/MTBE system, the reaction in the superabsorber/MTBE system was much slower. In the biphasic system, full conversion to alcohol (*R*)-**2** could be achieved after 5 h, whereas in the superabsorber system after 5 h only 60% conversion to (*R*)-**2** was observed. A maximum conversion to product (*R*)-**2** of 85% after 23 h was observed (Figure 5). Thereafter, no further conversion could be observed.

### 3.2. Studies on the Influence of Organic Solvents on the Reduction of Acetophenone with a Superabsorber-Immobilized Recombinant Alcohol Dehydrogenase from Lactobacillus brevis

To investigate the influence of different solvents, the organic phase was changed while other parameters were kept constant. A range of standard solvents with different properties were investigated. As dichloromethane (DCM) is usually not suitable for enzyme catalysis, it would be interesting to learn about the performance of the investigated system towards this solvent in comparison to other organic “standard solvents”. In addition, methyl *tert*-butyl ether (MTBE) and diethyl carbonate were chosen as greener solvent options.

**Figure 6:**
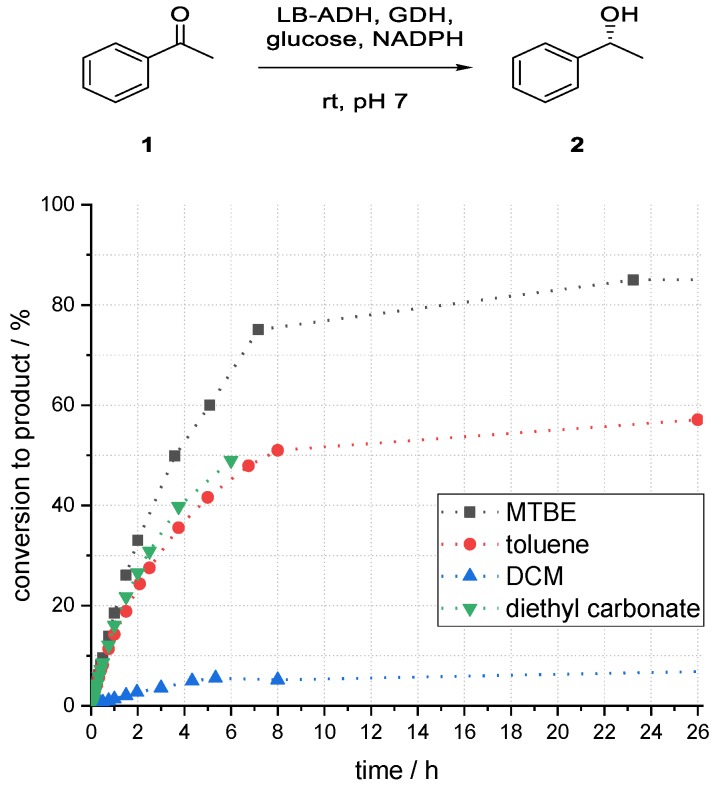
Influence of different solvents on the conversion to phenylethanol (*R*)-**2** in an enzymatic reduction of acetophenone (**1**) using ADH in superabsorber-immobilized aqueous phase.

As shown in Figure 6, the highest conversion to product (*R*)-**2** after 24 h was obtained for a system using MTBE as an organic solvent component (85%). A maximum conversion to the desired product of 55% for toluene could be observed after 26 h. Thereafter, no further product formation was observed. The result for diethyl carbonate was comparable to that of toluene. With DCM as solvent, the conversion of acetophenone to product (*R*)-**2** was with 10% even after 48 h reaction time very low. By far the best tested solvent was MTBE, which was therefore used for further experiments.

### 3.3. Studies on the Influence of Incubation Time on the Acetophenone Reduction with a Superabsorber-Immobilized Recombinant Alcohol Dehydrogenase from Lactobacillus brevis

A further investigation of the storage stability of the absorbed enzymes was carried out. For this purpose, the immobilized aqueous phase was incubated for 24 h at room temperature without organic solvent. 

**Figure 7:**
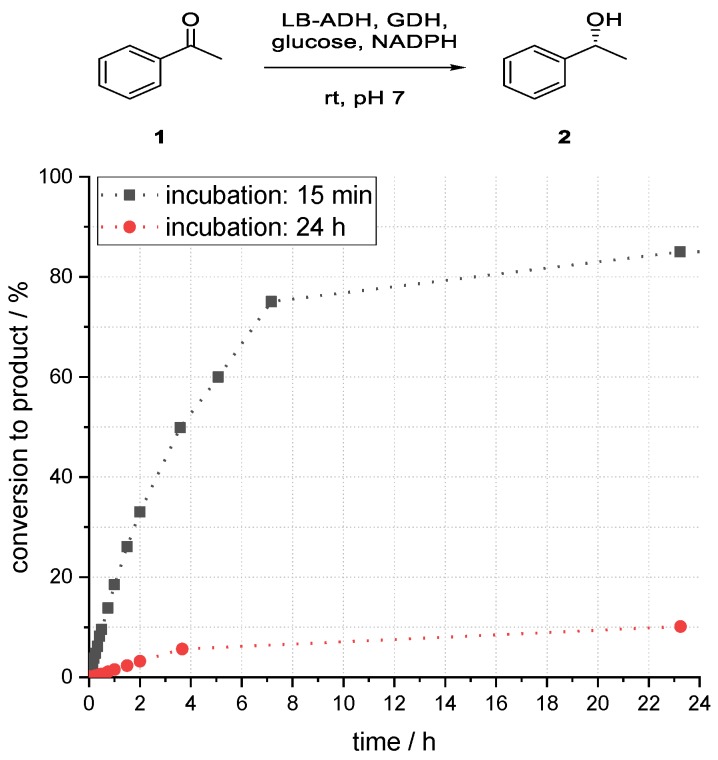
Influence of incubation time on the conversion to phenylethanol (*R*)-**2** in an enzymatic reduction of acetophenone (**1**) using ADH in superabsorber-immobilized aqueous phase.

It was found that the immobilized enzymes were not stable over a longer period. After incubation of the immobilized aqueous phase for 24 h, the maximum conversion to alcohol (*R*)-**2** after 23 h reaction time dropped from 85% to below 10%, as shown in Figure 7. Therefore, the storage stability of the immobilized enzymes was not very high. 

### 3.4. Studies on the Influence of the Ratio of Buffer to Solvent on the Acetophenone Reduction with a Superabsorber-Immobilized Recombinant Alcohol Dehydrogenase from Lactobacillus brevis

The influence of the ratio of the immobilized aqueous phase and organic phase was investigated. Change of phase interface area, distribution of compounds, as well as the enzyme environment can influence the performance of the system.

**Figure 8:**
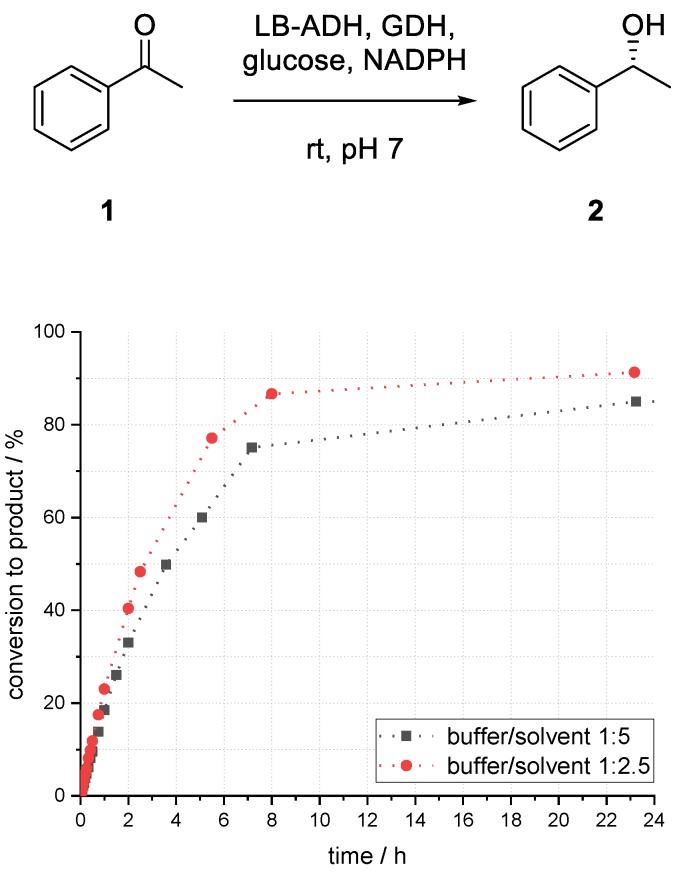
Influence of buffer-to-solvent ratio on the conversion to phenylethanol (*R*)-**2** in an enzymatic reduction of acetophenone (**1**) using ADH in superabsorber-immobilized aqueous phase.

By changing the aqueous to organic phase ratio from 1:5 to 1:2.5, an increase of conversion to product (*R*)-**2** after 23 h from 85% to 90% could be observed (Figure 8). Higher enzyme stability due to less direct contact of enzyme and solvent, and more favorable compound distribution might be the reasons for higher product formation.

### 3.5. Studies on the Influence of the Buffer Concentration on the Acetophenone Reduction with a Superabsorber-Immobilized Recombinant Alcohol Dehydrogenase from Lactobacillus brevis

Also, the influence of potassium phosphate buffer (PPB) buffer concentration was investigated. Because the superabsorber itself acts as a buffer system and because stoichiometric amounts of acid were formed due to the GDH-based cofactor regeneration system, this system was difficult to quantify.

**Figure 9:**
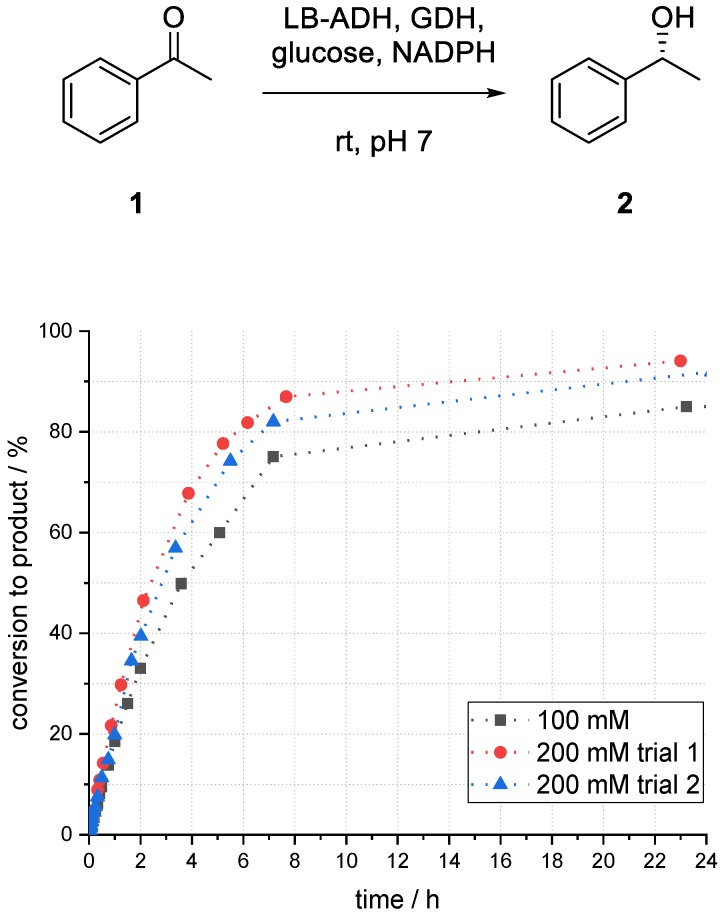
Influence of PPB buffer concentration on the conversion to phenylethanol (*R*)-**2** in an enzymatic reduction of acetophenone (**1**) using ADH in superabsorber-immobilized aqueous phase.

By increasing the PPB buffer concentration from 100 to 200 mM, the conversion to the desired product after 24 h increased from 85% to 94% (Figure 9). The results have been confirmed in a second experiment (92%). 

### 3.6. Studies on the Influence of the Superabsorber Particle Size on the Acetophenone Reduction with a Superabsorber-Immobilized Recombinant Alcohol Dehydrogenase from Lactobacillus brevis

Another process parameter of potential relevance was the interface area of organic and aqueous phase. By powdering the superabsorber granulate, the interface area, and therefore the mass transfer phenomena, should be significantly increased. For the preparation of superabsorber powder (<100 µm), the superabsorber granulate was powdered by grinding in a mortar and sieving with a steel mesh.

**Figure 10:**
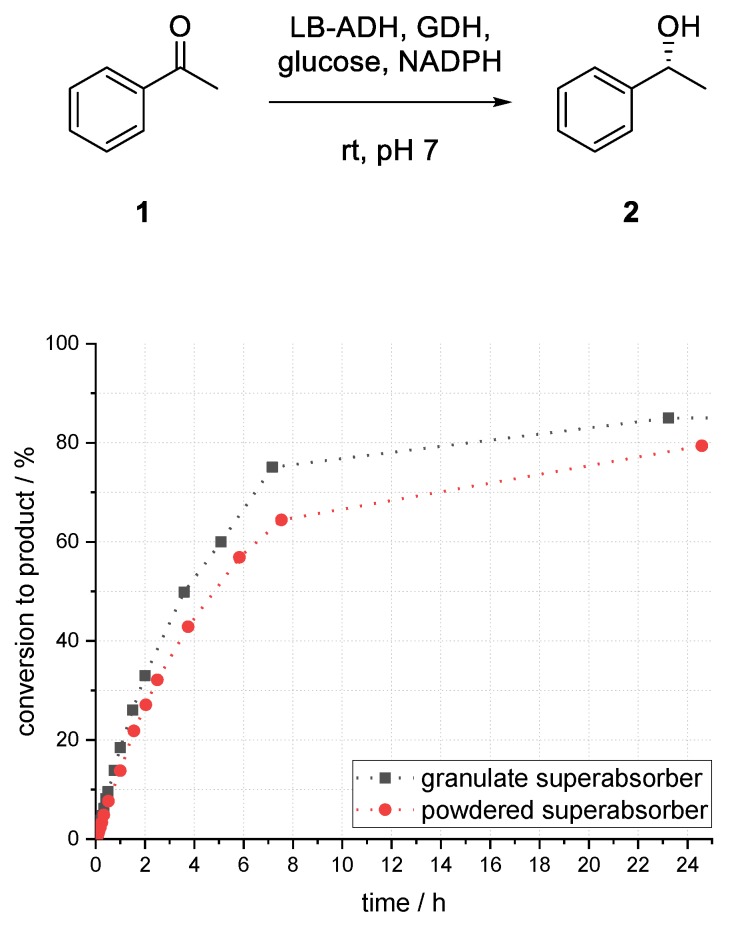
Influence of superabsorber particle size on the conversion to phenylethanol (*R*)-**2** in an enzymatic reduction of acetophenone (**1**) using ADH in superabsorber-immobilized aqueous phase.

As shown in Figure 10, a smaller particle size led to lower conversion. After 25 h, 6% less product (*R*)-**2** was formed compared to larger particle size after 23 h. Considering that the surface of the superabsorber particles was increased, phase transfer was not a limiting factor for this system. Due to smaller aqueous superabsorber particles, the solvent played a major role in deactivation of enzymes, as enzymes might be more often near to the surface.

### 3.7. Studies on the Influence of the Biocatalyst Loading on the Acetophenone Reduction with a Superabsorber-Immobilized Recombinant Alcohol Dehydrogenase from Lactobacillus brevis

Finally, the ADH loading was increased form 2 U mL^−1^ to 4 U mL^−1^. The amount of cofactor regeneration system was adjusted accordingly.

**Figure 11:**
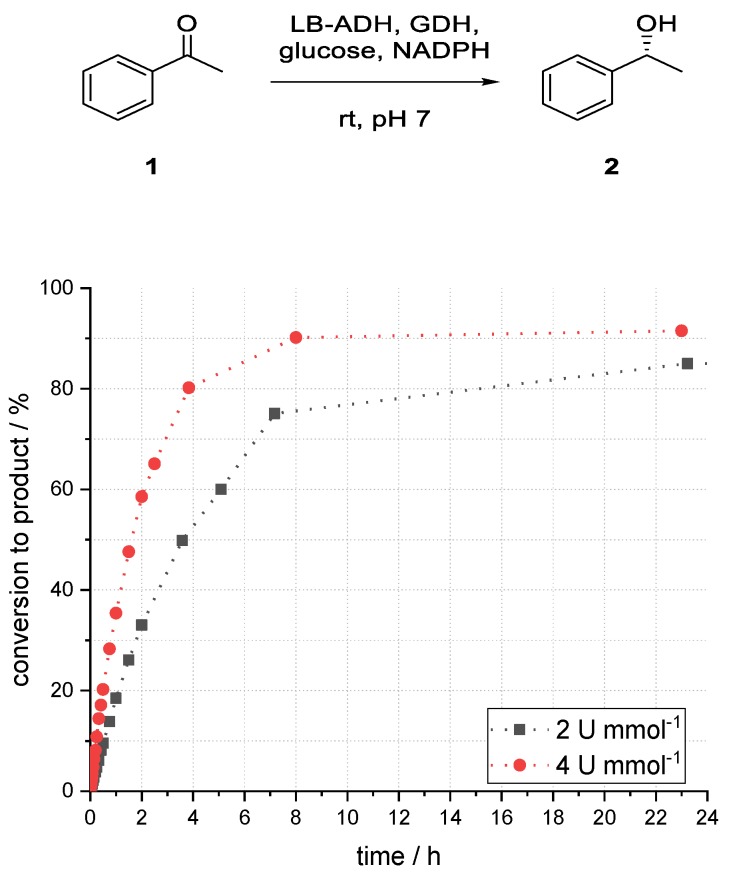
Influence of catalyst loading (in U mL^−1^) on the conversion to phenylethanol (*R*)-**2** in an enzymatic reduction of acetophenone (**1**) using ADH in superabsorber-immobilized aqueous phase.

As expected, the reaction using more catalyst performed better (85% vs. 92% after 23 h, Figure 10). This result also supported the assumption that mass transfer was not a limiting factor in the reaction system, as the conversion can be increased using more catalysts. Enzyme deactivation, however, was likely to have played a major role in this reaction because at lower catalyst loading (2 U mL−1) prolonging the reaction time after 8 h, no significant further increase of conversion could be observed (Figure 11). 

As for the tested solvents, it could be shown that MTBE was the best performing one. As expected, solvents that have previously shown to be not tolerated by enzymes, such as DCM, turned out not to be suitable for the investigated reaction system. MTBE and diethyl carbonate have both shown high conversions, indicating deactivation of the enzyme to a lesser extent. Besides stability issues, according to the solvent-dependent partition coefficient, substrate distribution in the aqueous and organic phases might also have played a role here. In the case of an unfavored partition coefficient (caused by a very high solubility of the substrate in the organic phase), the concentration of substrate in the aqueous superabsorber system might have been below the K_M_ value, thus slowing down the overall process.

### 3.8. Set-Up of a Packed Bed Reactor

**Figure 12:**
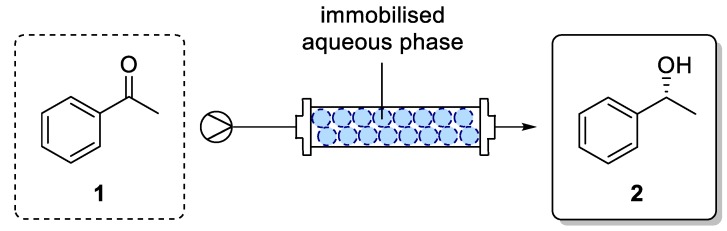
Schematic flow process of enzymatic reduction of acetophenone (**1**) to 1-phenylethanol (*R*)-**2** using superabsorber-immobilized ADH with GDH-based cofactor regeneration system in a packed bed reactor.

**Figure 13:**
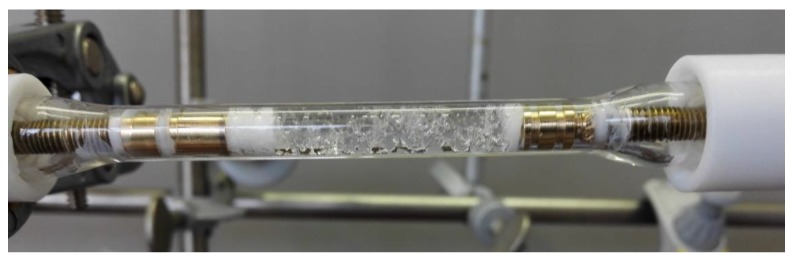
Developed glass reactor packed with superabsorber gel.

Figure 12 shows a schematic flow process for an enzymatic reduction of acetophenone (**1**) to phenylethanol (*R*)-**2**, coupled with a GDH-based cofactor regeneration system. The packed bed reactor was loaded with the immobilized aqueous phase, containing the enzymes, cofactor, and glucose. A total amount of 2 U ADH and a substrate concentration of 100 mM in MTBE were used for the experiments. For the setup, an in-house custom build glass tube reactor (ID 5 mm) was used (Figure 13).

The reactor was fed with a solution of ketone 1 in MTBE using a syringe pump. A residence time of 1 hour was set and collected fractions of the resulting product solution were analyzed by GC. Because the catalyst was immobilized, no separation or quenching of the reaction mixture was required.

### 3.9. Studies on the Acetophenone Reduction in a Flow Process with a Superabsorber-Immobilized Recombinant Alcohol Dehydrogenase from Lactobacillus brevis

**Figure 14:**
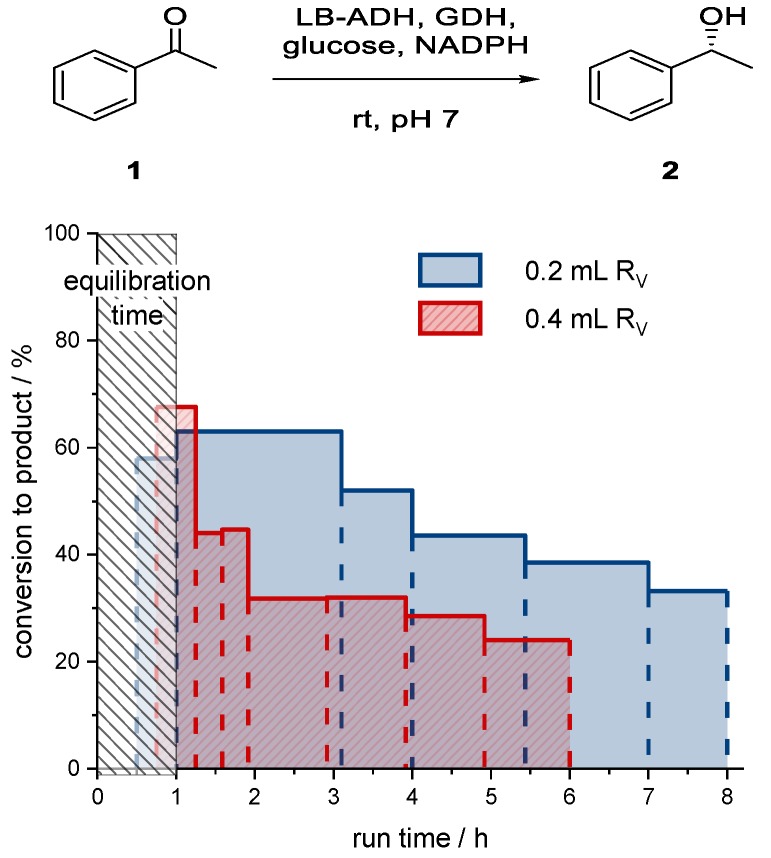
Conversion of acetophenone (**1**) to 1-phenylethanol (*R*)-**2** using superabsorber-immobilized ADH with a co-immobilized GDH-based cofactor regeneration system in a packed bed reactor. Horizontal lines show conversion for fractions collected between times indicated with dashed vertical lines. Reactor volume: 0.2 mL or 0.4 mL (glass reactor, inner diameter: 5 mm), residence time: 1 h, catalyst loading: 2 U, temperature: room temperature, c_substr_(organic): 100 mM.

The reactor was charged with different volumes of immobilized aqueous phase. The amounts of superabsorber and catalytic system (ADH, GDH, glucose, NADPH) were kept constant, whereas different volumes of buffer were used to adjust the overall reactor bed volume (R_V_). After charging of the reactor, the end pieces of the reactor were adjusted to the catalyst bed. The average conversions of collected fractions are shown in Figure 14.

As for the larger reactor volume, the initial conversion to alcohol (*R*)-**2** in the first hour was very high (67%). However, the conversion decreased very quickly to only 24% after 5 h. In the case of the smaller reactor bed volume, the conversion to alcohol (*R*)-**2** for the first fractions collected from the start to the first hour was about 61%. However, the conversion was not constant over a longer period, and dropped to 33% after 7 h of reactor operating time (or seven residence times). Compared to the larger reactor volume, the system was more stable. The smaller system was probably more stable due to lower flow rates and therefore less organic solvent pumped through the reactor bed. In accordance with the batch experiments, the stability of the catalyst was a major issue.

Compared to batch processes, a much higher initial conversion within 1 h reaction time could be achieved. Due to the nature of a packed bed reactor, the catalyst could be used more efficiently, as the catalyst concentration in the reaction was much higher (a summary can be found in Table 3).

It was successfully shown that a superabsorber-immobilisate can be applied in a packed bed reactor, thus offering an alternative to the standard approach using carrier-immobilized biocatalysts.

## 4. Conclusions

In conclusion, different factors have shown to be more or less important for the investigated reduction of acetophenone (**1**) in the presence of the LB-ADH (summarized in Table 4). The choice of solvent turned out to represent a crucial issue for this process. Although MTBE provided high conversions, DCM, for instance, led to deactivation of the catalytic system under the same reaction conditions. Mass transfer seemed to not be a limiting factor for this system under the investigated conditions.

Furthermore, the application of superabsorber-immobilized LB-ADH with a cofactor regeneration system in a packed bed reactor, thus running in a flow mode, was demonstrated. However, the stability of the immobilized enzymes under the investigated conditions was shown to be insufficient. Nevertheless, very high initial conversions for the PBR setup were observed. Further process development and extension of this PBR methodology with superabsorber-based redox enzymes towards other applications in biocatalytic ketone reduction and related redox biotransformations are currently in progress. In addition, in general (and thus, also for other types of enzymes) this type of PBR methodology represents an alternative to the use of standard carrier- immobilized biocatalysts, and this system also offers the perspective for applications beyond isolated enzymes and, for example, for whole cell catalysts [51].

## Figures and Tables

**Table 1:** Composition of the Bradford reagent.

Compound	Composition
Coomassie Blue G 250	50 mg mL^−1^ in methanol
*ortho*-phosphoric acid	100 mL
water	to 1 L

**Table 2:** Solutions for the photometer assay.

Compound	Composition	Volume/mL
Potassium phosphate buffer (PPB)	100 mM aq. soln., pH 7	446
acetophenone	22 mM in potassium phosphate buffer (100 mM aqueous solution, pH 7)	500
NADPH	10 mM aqueous solution	24
ADH crude extract	different dilutions	30

**Table 3:** Comparison of different reaction parameters and results obtained from flow and batch experiments (see Section 3.4 and Section 3.8). In all experiments, the same total amount of catalyst was used.

	Batch	Flow	
Reactor volume	2 mL	0.2 mL	0.4 mL
Flow rate	-	0.2 mL h^−1^	0.4 mL h^−1^
Reaction/residence time	1 h	1 h	1 h
Max conversion	23%	63% ^1^	45% ^1^
Selectivity	100%	100%	100%
Productivity ^2^	2.8 mg h^−1^	1.5 mg h^−1^	2.2 mg h^−1^
Space time yield	1.4 mg h^−1^ mL^−1^	7.5 mg h^−1^ mL^−1^	5.5 mg h^−1^ mL^−1^

^1^ For initial conversion of the operating time (0.2 mL R_V_: 60–190 min (three residence times); 0.4 mL R_V_: 45–75 min (one-quarter residence time). ^2^ Considering maximum conversion.

**Table 4:** Influence of different parameters on the reduction of acetophenone (**1**) in a biphasic superabsorber/MTBE system, considering conversions to alcohol (*R*)-**2** after approximately 24 h in comparison to an initial standard experiment (Section 3.1). Legend: − negative impact, + positive impact, parentheses indicate small impact.

Parameter	Influence	Relative Conversion
solvent (Section 3.2)	-	up to −75%
longer incubation time (Section 3.3)	-	−75%
higher superabsorber-solvent ratio (Section 3.4)	(+)	+7%
higher buffer concentration (Section 3.5)	(+)	+8%
higher catalyst loading (Section 3.7)	(+)	+7%
smaller particle size (Section 3.6)	(−)	−6%

## Appendix A

### Appendix A.1. GC Analytics

GC analysis was made using a GC-2010 Plus by Shimadzu, equipped with an AOC-20i autoinjector, a flame ionization detector (FID), and N_2_ carrier gas. Conversions were determined on the basis of calibration measurements of the compounds.

Column: chiral BGB-174 (0.25 mm ID, 0.25 µm film, 30 m length) by BGB Analytik AG.

Column oven temperature program: 100 °C to 110 °C with 1 °C min^−1^.

Settings: SPL1: 220 °C, pressure: 161.7 kPa, total flow: 26.2 mL min^−1^, column flow: 2.11 mL min^−1^, linear velocity: 46.9 cm s^−1^, purge flow: 3.0 mL min^−1^, split ratio: 10.0, FID: 220 °C.

Retention times: Acetophenone (**1**) 6.18 min, (*R*)-1-phenylethanol ((*R*)-**2**) 7.90 min, (*S*)-1-phenylethanol ((*S*)-1) 8.24 min.

### Appendix A.2. NMR Analytics

^1^H NMR spectra were recorded on a Bruker Avance III 500 spectrometer (500 MHz) in chloroform-d3, referenced internally to the residual solvent peaks ppm (chloroform-d3: 7.26 ppm (s)) and analyzed using MestReNova. Chemical shifts *δ* were reported in ppm to the nearest 0.01 ppm. The multiplicity of ^1^H signals were indicated as follows: s = singlet, d = doublet, dd = doublet of doublet, t = triplet, q = quadruplet, m = multiplet, br = broad, or combinations thereof. Coupling constants (*J*) were reported in Hz to the nearest 0.1 Hz.

### Appendix A.3. Standard Procedure 1 (SP1): Synthesis of R-1-Phenylethanol ((R)-2) Using LB-ADH in Batch Mode

In a glass vial, LB-ADH (2 U), GDH (6 U), glucose (c_final_(overall): 150 mM, 300 µL of 1 M in potassium phosphate buffer (100 mM, pH 7)), NADPH (c_final_(overall): 0.1 mM, 20 µL of 10 mM aqueous solution), and potassium phosphate buffer (to 2 mL, 100 mM, pH 7) were mixed. Then, acetophenone (1, c_final_(overall): 50 mM, 12.7 µL) in MTBE (1 mL) was added, and the reaction mixture was stirred at 25 °C. The conversion was analyzed via GC.

**^1^H NMR** (500 MHz, CDCl_3_): *δ* [ppm] = 7.43–7.35 (m, 4H, Ar-*H*), 7.33–7.28 (m, 1H, Ar-*H*), 4.92 (q, ^3^*J* = 6.5 Hz, 1H, CH_3_-C-*H*), 1.92 (br s, 1H, O-*H*), 1.53 (d, ^3^*J* = 6.4 Hz, 3H, -*CH*_3_).

**GC:** Retention times: acetophenone (**1**) 6.18 min, (*R*)-1-phenylethanol ((*R*)-**2**) 7.90 min, (*S*)-1-phenylethanol ((*S*)-1) 8.24 min.

**Table A1:** Reduction of acetophenone (**1**) to 1-phenylethanol (*R*)-**2**. Change from standard procedure: none.

Entry	Time	Conversion/%
1	2.5 min	1
2	4 min	1
3	6 min	2
4	10 min	5
5	15 min	8
6	20 min	9
7	30 min	15
8	45 min	24
9	60 min	32
10	1 h 30 min	45
11	1 h 52 min	51
12	3 h 50 min	69
13	5 h 16 min	79
14	24 h	89

**Table A2:** Reduction of acetophenone (**1**) to 1-phenylethanol (*R*)-**2**. Change from standard procedure: buffer (to 2 mL), MTBE (1668 µL).

Entry	Time	Conversion/%
1	1 min	3
2	2 min	4
3	3 min	6
4	4 min	7
5	5 min	8
6	7 min	11
7	9 min	14
8	12 min	17
9	15 min	21
10	20 min	27
11	25 min	32
12	30 min	37
13	45 min	49
14	60 min	59
15	1 h 30 min	75
16	2 h	87
17	5 h	100

### Appendix A.4. Standard Procedure 2 (SP2): Synthesis of 1-Phenylethanol (R)-2 Using Superabsorber-Immobilized Enzymes

In a glass vial, LB-ADH (2 U), GDH (6 U), glucose (c_final_(overall): 150 mM, 300 µL of 1 M in potassium phosphate buffer (100 mM, pH 7)), and NADPH (c_final_(overall): 0.1 mM, 20 µL of 10 mM aqueous solution) were mixed. Then, superabsorber (20 mg, Favor SXM 9155) was added to the colorless solution. After all liquid was absorbed, a solution of acetophenone (1, c_final_(overall): 50 mM, 11.7 µL) in MTBE (1668 µL) was added. The mixture was stirred at 25 °C at 800 rpm. The conversion was analyzed via GC.

**^1^H NMR** (500 MHz, CDCl_3_): *δ* (ppm) = 7.43–7.35 (m, 4H, Ar-*H*), 7.33–7.28 (m, 1H, Ar-*H*), 4.92 (q, ^3^*J* = 6.5 Hz, 1H, CH_3_-C-*H*), 1.92 (br s, 1H, O-*H*), 1.53 (d, ^3^*J* = 6.4 Hz, 3H, -C*H*_3_).

**GC:** Retention times: acetophenone (**1**) 6.18 min, (*R*)-1-phenylethanol ((*R*)-**2**) 7.90 min, (*S*)-1-phenylethanol ((*S*)-1) 8.24 min.

**Table A3:** Reduction of acetophenone (**1**) to 1-phenylethanol (*R*)-**2**. Change from standard procedure: none.

Entry	Time	Conversion/%
1	1 min	0
2	2 min	0
3	3 min	1
4	4 min	1
5	5 min	2
6	7 min	2
7	9 min	3
8	12 min	4
9	15 min	5
10	20 min	6
11	25 min	8
12	30 min	10
13	45 min	14
14	60 min	19
15	1 h 30 min	26
16	2 h 0 min	33
17	3 h 35 min	50
18	5 h 5 min	60
19	7 h 10 min	75
20	23 h 14 min	85
21	46 h 54 min	86
22	70 h 54 min	86
23	94 h 24 min	86

**Table A4:** Reduction of acetophenone (**1**) to 1-phenylethanol (*R*)-**2**. Change from standard procedure: toluene (solvent).

Entry	Time	Conversion/%
1	1 min	0
2	2 min	1
3	3 min	1
4	4 min	1
5	5 min	1
6	7 min	2
7	9 min	3
8	12 min	4
9	15 min	4
10	20 min	6
11	25 min	7
12	30 min	8
13	45 min	11
14	60 min	14
15	1 h 30 min	19
16	2 h 5 min	24
17	2 h 30 min	28
18	3 h 45 min	36
19	5 h 0 min	42
20	6 h 45 min	51
21	8 h 0 min	57
22	26 h 0 min	57
23	31 h 0 min	57
24	50 h 30 min	58
25	74 h 30 min	58
26	98 h 30 min	58
27	121 h 40 min	58

**Table A5:** Reduction of acetophenone (**1**) to 1-phenylethanol (*R*)-**2**. Change from standard procedure: dichloromethane (DCM) (solvent).

Entry	Time	Conversion/%
1	1 min	0
2	2 min	0
3	3 min	0
4	4 min	0
5	5 min	0
6	7 min	0
7	9 min	0
8	12 min	0
9	15 min	0
10	20 min	0
11	25 min	1
12	30 min	1
13	45 min	1
14	60 min	1
15	1 h 30 min	2
16	1 h 58 min	3
17	3 h 0 min	4
18	4 h 20 min	5
19	5 h 20 min	6
20	8 h 0 min	5
21	27 h 19 min	7
22	31 h 49 min	9
23	48 h 19 min	10
24	72 h 19 min	10
25	96 h 19 min	10
26	119 h 49 min	10

**Table A6:** Reduction of acetophenone (**1**) to 1-phenylethanol (*R*)-**2**. Change from standard procedure: diethyl carbonate (solvent).

Entry	Time	Conversion/%
1	1 min	0
2	2 min	1
3	3 min	1
4	4 min	1
5	5 min	1
6	7 min	2
7	9 min	3
8	12 min	4
9	15 min	4
10	20 min	6
11	25 min	7
12	30 min	9
13	45 min	12
14	60 min	16
15	1 h 30 min	22
16	2 h 0 min	27
17	2 h 30 min	31
18	3 h 45 min	40
19	6 h 0 min	49

**Table A7:** Reduction of acetophenone (**1**) to 1-phenylethanol (*R*)-**2**. Change from standard procedure: 24 h incubation of immobilized enzymes in superabsorber without organic solvent.

Entry	Time	Conversion/%
1	1 min	0
2	2 min	0
3	3 min	0
4	12 min	0
5	15 min	0
6	20 min	0
7	25 min	1
8	30 min	1
9	45 min	1
10	60 min	2
11	1 h 30 min	2
12	2 h 0 min	3
13	3 h 40 min	6
14	23 h 15 min	10

**Table A8:** Reduction of acetophenone (**1**) to 1-phenylethanol (*R*)-**2**. Change from standard procedure: buffer/solvent ratio 1:2.5 instead of 1:5, adjusted by solvent volumes.

Entry	Time	Conversion/%
1	1 min	0
2	2 min	1
3	3 min	1
4	4 min	2
5	5 min	2
6	7 min	3
7	9 min	3
8	12 min	5
9	15 min	6
10	20 min	8
11	25 min	10
12	30 min	12
13	45 min	18
14	60 min	23
15	2 h 0 min	40
16	2 h 30 min	48
17	5 h 30 min	77
18	8 h 0 min	87
19	23 h 10 min	91

**Table A9:** Reduction of acetophenone (**1**) to 1-phenylethanol (*R*)-**2**. Change from standard procedure: doubled enzyme and cofactor loadings.

Entry	Time	Conversion/%
1	1 min	1
2	2 min	1
3	3 min	2
4	4 min	3
5	5 min	4
6	7 min	5
7	9 min	6
8	12 min	8
9	15 min	11
10	20 min	14
11	25 min	17
12	30 min	20
13	45 min	28
14	60 min	35
15	1 h 30 min	48
16	2 h 0 min	59
17	3 h 30 min	65
18	3 h 50 min	80
19	8 h 0 min	90
20	23 h 0 min	92

**Table A10:** Reduction of acetophenone (**1**) to 1-phenylethanol (*R*)-**2**. Change from standard procedure: 200 mM potassium phosphate buffer trial 1.

Entry	Time	Conversion/%
1	1 min	1
2	2 min	1
3	3 min	1
4	4 min	1
5	5 min	2
6	7 min	3
7	9.5 min	4
8	12 min	5
9	20 min	9
10	25 min	11
11	33 min	14
12	52 min	22
13	1 h 15 min	30
14	2 h 7 min	47
15	3 h 52 min	68
16	5 h 13 min	78
17	6 h 10 min	82
18	7 h 40 min	87
19	23 h 0 min	94

**Table A11:** Reduction of acetophenone (**1**) to 1-phenylethanol (*R*)-**2**. Change from standard procedure: 200 mM potassium phosphate buffer trial 2.

Entry	Time	Conversion/%
1	1 min	0
2	2 min	1
3	3 min	1
4	4 min	1
5	5 min	2
6	7 min	3
7	9 min	3
8	12 min	5
9	15 min	6
10	20 min	8
11	30 min	11
12	45 min	15
13	60 min	20
14	1 h 39 min	35
15	2 h 01min	39
16	3 h 22 min	57
17	5 h 30 min	74
18	7 h 10 min	82
19	24 h 12 min	92

**Table A12:** Reduction of acetophenone (**1**) to 1-phenylethanol (*R*)-**2.** Change from standard procedure: superabsorber (100 mg), LB-ADH (46.9 µL), GDH (159.5 µL), NADPH (100 µL), glucose (1.5 mL), MTBE (3 mL), acetophenone (58.3 µL).

Entry	Time	Conversion/%
1	1 min	0
2	2 min	1
3	3 min	1
4	4 min	2
5	5 min	2
6	7 min	4
7	9 min	5
8	12 min	6
9	15 min	8
10	20 min	11
11	25 min	13
12	31 min	17
13	45 min	34
14	60 min	30
15	1 h 33	43
16	2 h 0 min	53
17	3 h 10 min	68
18	4 h 52 min	81
19	6 h 12 min	86
20	7 h 10 min	88
21	8 h 40 min	89
22	24 h 0 min	92

**Table A13:** Reduction of acetophenone (**1**) to 1-phenylethanol (*R*)-**2**. Change from standard procedure: powdered superabsorber (particle size < 100 µm).

Entry	Time	Conversion/%
1	1 min	0
2	2 min	0
3	3 min	0
4	4 min	1
5	5 min	1
6	7 min	1
7	9 min	2
8	12 min	2
9	15 min	3
10	20 min	5
11	31 min	8
12	60 min	14
13	1 h 33 min	22
14	2 h 2 min	27
15	2 h 30 min	32
16	3 h 45 min	43
17	5 h 0 min	57
18	7 h 32min	64
19	24 h 35 min	79

### Appendix A.5. Standard Procedure 3 (SP3): Synthesis of 1-Phenylethanol (R)-2 Using Superabsorber-Immobilized Enzymes

A glass reactor was filled with a layer of cotton (5 mm), then superabsorber (35 mg, Favor SXM 9155) was added. A solution of LB-ADH (crude extract in potassium phosphate buffer, 9.4 µL, 320 U mL^−1^), GDH (crude extract in potassium phosphate buffer, 31.9 µL, 188 U mL^−1^), glucose (300 µL of 1 M solution in potassium phosphate buffer (100 mM, pH 7)), and NADPH (20 µL of 10 mM solution in water) in potassium phosphate buffer (1.5 mL, 100 mM, pH 7) was pumped into the reactor to be immobilized at room temperature for 30 min. The reactor was filled with an additional cotton layer (5 mm) and sealed. A solution of acetophenone (35.1 µL, 0.3 mmol) in MTBE (3 mL) was transferred into a syringe (5 mL, from S.G.E. analytics, gas tight, 10.3 mm ID), which was attached to a syringe pump. The pump was connected to the reactor and set to a flow rate of 0.2 mL min^−1^ corresponding to a residence time of 1 h. Fractions were collected, and the conversion was determined via GC analysis.

**^1^H NMR** (500 MHz, CDCl_3_): *δ* (ppm) = 7.43–7.35 (m, 4H, Ar-*H*), 7.33–7.28 (m, 1H, Ar-*H*), 4.92 (q, ^3^*J* = 6.5 Hz, 1H, CH_3_-C-*H*), 1.92 (br s, 1H, O-*H*), 1.53 (d, ^3^*J* = 6.4 Hz, 3H, -C*H*_3_).

**GC:** Retention times: acetophenone (**1**) 6.18 min, (*R*)-1-phenylethanol ((*R*)-**2**) 7.90 min, (*S*)-1-phenylethanol ((*S*)-1) 8.24 min.

**Table A14:** Reduction of acetophenone (**1**) to 1-phenylethanol (*R*)-**2**. Change from standard procedure: none.

Entry	Fraction Time/min	Conversion/%
1	45–75	68
2	75–95	44
3	95–115	45
4	115–175	32
5	175–235	32
6	235–295	29
7	295–360	24

**Table A15:** Reduction of acetophenone (**1**) to 1-phenylethanol (*R*)-**2**. Change from standard procedure: glucose (900 µL), superabsorber (60 mg, resulting in 0.4 mL reactor bed volume, flow rate: 0.4 mL min^−1^).

Entry	Fraction Time/min	Conversion/%
1	30–60	58
2	60–186	63
3	186–240	52
4	240–326	44
5	326–420	39
6	420–480	33

### Appendix A.6. Gene and Amino Acid Sequence of the Alcohol Dehydrogenase from Lactobacillus brevis

References [46,47].

(PDB: 1NXQ)

Vector: pET21

Nucleic acid sequence:

ATGTCTAACCGTTTGGATGGTAAGGTAGCAATCATTACAGGTGGTACGTTGGGTATCGGTTTAGCTATCGCCACGAAGTTCGTTGAAGAAGGGGCTAAGGTCATGATTACCGGCCGGCACAGCGATGTTGGTGAAAAAGCAGCTAAGAGTGTCGGCACTCCTGATCAGATTCAATTTTTCCAACATGATTCTTCCGATGAAGACGGCTGGACGAAATTATTCGATGCAACGGAAAAAGCCTTTGGCCCAGTTTCTACATTAGTTAATAACGCTGGGATCGCGGTTAACAAGAGTGTCGAAGAAACCACGACTGCTGAATGGCGTAAATTATTAGCCGTCAACCTTGATGGTGTCTTCTTCGGTACCCGATTAGGGATTCAACGGATGAAGAACAAAGGCTTAGGGGCTTCCATCATCAACATGTCTTCGATCGAAGGCTTTGTGGGTGATCCTAGCTTAGGGGCTTACAACGCATCTAAAGGGGCCGTACGGATTATGTCCAAGTCAGCTGCCTTAGATTGTGCCCTAAAGGACTACGATGTTCGGGTAAACACTGTTCACCCTGGCTACATCAAGACACCATTGGTTGATGACCTACCAGGGGCCGAAGAAGCGATGTCACAACGGACCAAGACGCCAATGGGCCATATCGGTGAACCTAACGATATTGCCTACATCTGTGTTTACTTGGCTTCTAACGAATCTAAATTTGCAACGGGTTCTGAATTCGTAGTTGACGGTGGCTACACTGCTCAATAG

Amino acid sequence:

MSNRLDGKVAIITGGTLGIGLAIATKFVEEGAKVMITGRHSDVGEKAAKSVGTPDQIQFFQHDSSDEDGWTKLFDATEKAFGPVSTLVNNAGIAVNKSVEETTTAEWRKLLAVNLDGVFFGTRLGIQRMKNKGLGASIINMSSIEGFVGDPSLGAYNASKGAVRIMSKSAALDCALKDYDVRVNTVHPGYIKTPLVDDLPGAEEAMSQRTKTPMGHIGEPNDIAYICVYLASNESKFATGSEFVVDGGYTAQ

## References

[1] Ahmed-Omer B., Brandt J.C., Wirth T. (2007). Advanced organic synthesis using microreactor technology. Org. Biomol. Chem..

[2] Chatterjee S. FDA Perspective on Continuous Manufacturing. https://www.fda.gov/media/85366/download.

[3] Hernán D. Continuous manufacturing: Challenges and opportunities. EMA perspective. Proceedings of the 3rd FDA/PQRI Conference on Advancing Product Quality.

[4] Gutmann B., Cantillo D., Kappe C.O. (2015). Continuous-Flow Technology-A Tool for the Safe Manufacturing of Active Pharmaceutical Ingredients. Angew. Chem. Int. Ed..

[5] Movsisyan M., Delbeke E.I.P., Berton J.K.E.T., Battilocchio C., Ley S.V., Stevens C.V. (2016). Taming hazardous chemistry by continuous flow technology. Chem. Soc. Rev..

[6] Malet-Sanz L., Susanne F. (2012). Continuous flow synthesis. A pharma perspective. J. Med. Chem..

[7] Britton J., Majumdar S., Weiss G.A. (2018). Continuous flow biocatalysis. Chem. Soc. Rev..

[8] Tamborini L., Fernandes P., Paradisi F., Molinari F. (2018). Flow Bioreactors as Complementary Tools for Biocatalytic Process Intensification. Trends Biotechnol..

[9] Laurenti E., dos Santos Vianna A. (2016). Enzymatic microreactors in biocatalysis: History, features, and future perspectives. Biocatalysis.

[10] Sato H., Hummel W., Gröger H. (2015). Cooperative Catalysis of Noncompatible Catalysts through Compartmentalization: Wacker Oxidation and Enzymatic Reduction in a One-Pot Process in Aqueous Media. Angew. Chem. Int. Ed..

[11] Rudroff F., Mihovilovic M.D., Gröger H., Snajdrova R., Iding H., Bornscheuer U.T. (2018). Opportunities and challenges for combining chemo- and biocatalysis. Nat. Catal..

[12] Gröger H., Williams G., Hall M. (2018). Emerging Fields in One-pot Multi-step Synthesis with Combined Chemo- and Bio-catalysts: Sequential- and Domino-type Process Concepts as well as Compartmentation Strategies. Modern Biocatalysis: Advances Towards Synthetic Biological Systems.

[13] Goldberg K., Schroer K., Lütz S., Liese A. (2007). Biocatalytic ketone reduction—A powerful tool for the production of chiral alcohols—Part I: Processes with isolated enzymes. Appl. Microbiol. Biotechnol..

[14] Moore J.C., Pollard D.J., Kosjek B., Devine P.N. (2007). Advances in the Enzymatic Reduction of Ketones. Acc. Chem. Res..

[15] Mayr J.C., Grosch J., Hartmann L., Rosa L.F.M., Spiess A.C., Harnisch F. (2019). Resting Escherichia coli as Chassis for Microbial Electrosynthesis: Production of Chiral Alcohols. ChemSusChem.

[16] Thorey P., Knez Ž., Habulin M. (2010). Alcohol dehydrogenase in non-aqueous media using high-pressure technologies: Reaction set-up and deactivation determination. J. Chem. Technol. Biotechnol..

[17] Bordón D.L., Villalba L.D., Aimar M.L., Cantero J.J., Vázquez A.M., Formica S.M., Krapacher C.R., Rossi L.I. (2015). Weeds as biocatalysts in the stereoselective synthesis of chiral phenylethanols used as key intermediates for pharmaceuticals. Biocatal. Agric. Biotechnol..

[18] Ramos T.S., Luz D.M., Nascimento R.D., Silva A.K., Lião L.M., Miranda V.M., Deflon V.M., de Araujo M.P., Ueno L.T., Machado F.B.C. (2019). Ruthenium-cymene containing pyridine-derived aldiimine ligands: Synthesis, characterization and application in the transfer hydrogenation of aryl ketones and kinetics studies. J. Organomet. Chem..

[19] Wang B., Zhang C., Wang J., An H., Guo Z., Lv Z. (2019). Study of nano-Cu/SiO_2_ catalysts for highly selective hydrogenation of acetophenone. Appl. Organomet. Chem..

[20] Lee J.K., Samanta D., Nam H.G., Zare R.N. (2019). Micrometer-Sized Water Droplets Induce Spontaneous Reduction. J. Am. Chem. Soc..

[21] Bondue C.J., Koper M.T.M. (2019). Electrochemical Reduction of the Carbonyl Functional Group: The Importance of Adsorption Geometry, Molecular Structure, and Electrode Surface Structure. J. Am. Chem. Soc..

[22] Thompson M.P., Peñafiel I., Cosgrove S.C., Turner N.J. (2019). Biocatalysis Using Immobilized Enzymes in Continuous Flow for the Synthesis of Fine Chemicals. Org. Process Res. Dev..

[23] Peschke T., Bitterwolf P., Gallus S., Hu Y., Oelschlaeger C., Willenbacher N., Rabe K.S., Niemeyer C.M. (2018). Self-Assembling All-Enzyme Hydrogels for Flow Biocatalysis. Angew. Chem. Int. Ed..

[24] Hartley C.J., Williams C.C., Scoble J.A., Churches Q.I., North A., French N.G., Nebl T., Coia G., Warden A.C., Simpson G. (2019). Engineered Enzymes that Retain and Regenerate their Cofactors Enable Continuous-Flow Biocatalysis. Nat. Catal..

[25] Döbber J., Pohl M., Ley S.V., Musio B. (2018). Rapid, selective and stable HaloTag-Lb ADH immobilization directly from crude cell extract for the continuous biocatalytic production of chiral alcohols and epoxides. React. Chem. Eng..

[26] Brahma A., Musio B., Ismayilova U., Nikbin N., Kamptmann S.B., Siegert P., Jeromin G.E., Ley S.V., Pohl M. (2016). An orthogonal biocatalytic approach for the safe generation and use of HCN in a multistep continuous preparation of chiral O-acetylcyanohydrins. Synlett.

[27] Tang X., Allemann R.K., Wirth T. (2017). Optimising Terpene Synthesis with Flow Biocatalysis. Eur. J. Org. Chem..

[28] Liese A., Zelinski T., Kula M.-R., Kierkels H., Karutz M., Kragl U., Wandrey C. (1998). A novel reactor concept for the enzymatic reduction of poorly soluble ketones. J. Mol. Catal. B Enzym..

[29] Haberland J., Hummel W., Daußmann T., Liese A. (2002). New continuous production process for enantiopure (2R,5R)-hexanediol. Org. Process Res. Dev..

[30] Hildebrand F., Lütz S. (2006). Immobilisation of alcohol dehydrogenase from Lactobacillus brevis and its application in a plug-flow reactor. Tetrahedron Asymmetry.

[31] Šalić A., Pindrić K., Podrepšek G.H., Leitgeb M., Zelić B. (2013). NADH oxidation in a microreactor catalysed by ADH immobilised on γ-Fe2O3nanoparticles. Green Process. Synth..

[32] Karande R., Schmid A., Buehler K. (2010). Enzyme catalysis in an aqueous/organic segment flow microreactor: Ways to stabilize enzyme activity. Langmuir.

[33] Karande R., Schmid A., Buehler K. (2011). Miniaturizing biocatalysis: Enzyme-catalyzed reactions in an aqueous/organic segmented flow capillary microreactor. Adv. Synth. Catal..

[34] Adebar N., Choi J.E., Schober L., Miyake R., Iura T., Kawabata H., Gröger H. (2019). Overcoming Work-Up Limitations of Biphasic Biocatalytic Reaction Mixtures Through Liquid-Liquid Segmented Flow Processes. ChemCatChem.

[35] Sheldon R.A., van Pelt S. (2013). Enzyme immobilisation in biocatalysis: Why, what and how. Chem. Soc. Rev..

[36] Rosevear A. (1984). Immobilised Biocatalysts—A Critical Review. J. Chem. Technol. Biotechnol..

[37] Sigurdardóttir S.B., Lehmann J., Ovtar S., Grivel J.C., Della Negra M., Kaiser A., Pinelo M. (2018). Enzyme Immobilization on Inorganic Surfaces for Membrane Reactor Applications: Mass Transfer Challenges, Enzyme Leakage and Reuse of Materials. Adv. Synth. Catal..

[38] Lee C., Sandig B., Buchmeiser M.R., Haumann M. (2018). Supported ionic liquid phase (SILP) facilitated gas-phase enzyme catalysis—CALB catalyzed transesterification of vinyl propionate. Catal. Sci. Technol..

[39] Uhrich D., von Langermann J. (2017). Preparation and characterization of enzyme compartments in UV-Cured polyurethane-based materials and their application in enzymatic reactions. Front. Microbiol..

[40] Sun Q., Fu C.-W., Aguila B., Perman J., Wang S., Huang H.-Y., Xiao F.-S., Ma S. (2018). Pore Environment Control and Enhanced Performance of Enzymes Infiltrated in Covalent Organic Frameworks. J. Am. Chem. Soc..

[41] Zdarta J., Meyer A.S., Jesionowski T., Pinelo M. (2018). Developments in support materials for immobilization of oxidoreductases: A comprehensive review. Adv. Colloid Interface Sci..

[42] Jeromin G.E. (2009). Superabsorbed alcohol dehydrogenase-a new catalyst for asymmetric reductions. Biotechnol. Lett..

[43] Jeromin G.E. (2011). Immobilisierung von Alkoholdehydrogenasen und Deren Coenzyme Sowie Verwendung des Immobilisats. German Patent.

[44] Heidlindemann M., Rulli G., Berkessel A., Hummel W., Gröger H. (2014). Combination of asymmetric organo- and biocatalytic reactions in organic media using immobilized catalysts in different compartments. ACS Catal..

[45] Furno F., Müller F., Peggau J., Keup M., Schmidt H. (2007). Geruchsbindende Superabsorbierende Zusammensetzung 2005. Patent.

[46] Hummel W. (1997). New alcohol dehydrogenases for the synthesis of chiral compounds. Adv. Biochem. Eng./Biotechnol..

[47] Riebel B. (1996). Biochemische und Molekularbiologische Charakterisierung Neuer Mikrobieller NAD(P)-abhängiger Alkoholdehydrogenasen. Ph.D. Thesis.

[48] Weckbecker A., Gröger H., Hummel W. (2010). Regeneration of nicotinamide coenzymes: Principles and applications for the synthesis of chiral compounds. Adv. Biochem. Eng. Biotechnol..

[49] Vázquez-Figueroa E., Chaparro-Riggers J., Bommarius A.S. (2007). Development of a Thermostable Glucose Dehydrogenase by a Structure-Guided Consensus Concept. ChemBioChem.

[50] Zumbrägel N., Merten C., Huber S.M., Gröger H. (2018). Enantioselective reduction of sulfur-containing cyclic imines through biocatalysis. Nat. Commun..

[51] Hinzmann A., Adebar N., Betke T., Jochmann M., Gröger H. (2019). Biotransformations in Pure Organic Medium: Organic Solvent-Labile Enzymes in the Batch and Flow Synthesis of Nitriles. Eur. J. Org. Chem..

